# Editorial: Cucurbitaceae: multi-omics, functional analysis, and molecular breeding

**DOI:** 10.3389/fpls.2023.1301212

**Published:** 2023-10-03

**Authors:** Qiusheng Kong, Yi Zheng, Jie Zhang, Yang Bai

**Affiliations:** ^1^ National Key Laboratory for Germplasm Innovation & Utilization of Horticultural Crops, College of Horticulture and Forestry Sciences, Huazhong Agricultural University, Wuhan, China; ^2^ College of Plant Science and Technology, Beijing University of Agriculture, Beijing, China; ^3^ Beijing Academy of Agricultural and Forestry Sciences, Beijing, China; ^4^ Rensselaer Polytechnic Institute, Troy, NY, United States

**Keywords:** Cucurbitaceae, multi-omics, functional analysis, molecular breeding, editorial

As the second largest vegetable group, cucurbits are important economic crops and enjoy great popularity worldwide. After a complex domestication history, there are now a wide variety of cucurbit vegetables, including watermelon, melon, pumpkin, cucumber, bitter melon, bitter gourd, wax gourd, and many other species. In recent years, climate change has exacerbated a series of environmental stresses, such as extreme temperatures, drought, salinity, pests and diseases, which would greatly affect global crop production. Increasing attention has been placed on diverse germplasm resources to explore potential varieties with favorable agronomic traits for cucurbit breeding. Technological advances have enabled a comprehensive knowledge of the physiological and biochemical processes, molecular regulation, and genetic information at the multi-omics level for Cucurbitaceae. To address these problems, this Research Topic focus on the latest findings to describe the complex molecular mechanisms controlling the growth, development, and stress tolerance in Cucurbitaceae plants from a systematic biology perspective. The main goal of this Research Topic is to shed light on the progress made in multi-omics, functional analysis, and molecular breeding for cucurbit crops.

Several loci controlling important traits were genetically mapped for cucurbit crops and the candidate genes were proposed. Nie et al. assembled the parental genomes of a F_2_ population and identified 15 quantitative trait loci (QTL) for fruit quality-related traits in watermelon using a high-density genetic map. Sequence variation analysis showed that a gene encoding phytoene synthase was the candidate gene regulating watermelon flesh color changes. Yi et al. found that white flesh trait of watermelon was controlled by a single recessive locus and referred to as *Clwf2*. They mapped the *Clwf2* locus to a 132.3 kb region on chromosome 6 and the gene encoding a tetratricopeptide repeat protein was proposed as the candidate gene. Zhan et al. constructed a recombinant inbred line population containing 164 lines and detected a major QTL controlling watermelon fruit cracking on chromosome 2 using a high-density genetic map. Gong et al. identified five significant single nucleotide polymorphisms closely associated with watermelon rind thickness on chromosome 2 by genome-wide association study and a MADS family gene was proposed as the candidate gene. Zhu et al. found that leaf yellowing of watermelon was controlled by a single recessive gene and mapped the controlling gene in a region of 2.217 Mb on chromosome 2. Moreover, Liu et al. constructed an interspecific high-density genetic linkage map for *Luffa acutangula* and *Luffa cylindrica* for the first time and identified 40 QTLs related to 25 important agronomic traits. These results provide valuable information for maker development and target gene cloning.

Several genes and pathways related to stress response were characterized for cucurbit crops. Zhang et al. identified 14 members of the *Mildew Locus O* (*MLO*) gene family across melon genome and found *CmMLO5* (*MELO3C012438*) gene played a negative role in regulating powdery mildew-resistance in the susceptible melon line. Cai et al. analyzed the defense responses of wax gourd to wilt disease caused by *Phytophthora melonis* infection and found that phenylpropanoid biosynthesis, plant-pathogen interaction, the mitogen-activated protein kinase signaling pathway and plant hormone signal transduction were the most relevant pathways during the response of wax gourd to *P. melonis* infection. Luo et al. identified seven *DELLA* genes in pumpkin and revealed their potential roles under development and abiotic stresses. These genes and pathways undoubtedly deepen our understanding on the resistance or tolerance of cucurbit crops to biotic and abiotic stresses.

Germplasm innovation was performed for cucurbit crops. Yu et al. used ethyl methanesulfonate to mutate 10,000 seeds to construct a mutant collection for bitter melon and identified 14 lines with mutated phenotypes. Yan et al. identified a cucumber albino *alc* mutant and found it was recessively controlled by a single locus. They further investigated the pathways changed in the albino mutant using transcriptome analysis. These novel germplasms provide essential resources for genetic studies and breeding of cucurbit crops.

Multi-omics approach was used to investigate the regulating mechanism of important traits. Xie et al. identified 31 differentially accumulated flavonoids and 828 differentially expressed genes between the *hfc12* mutant with light-color pericarp and wild-type ‘BWT’ of wax gourd. Then, they found that the light-color pericarp and higher flavonoid content was controlled by a single gene *BhiPRR6* (*Bhi12M000742*) by using bulked segregant analysis-seq and kompetitive allele specific PCR analysis. Deng et al. explored the molecular fluctuations in pumpkin leaves at different time intervals after foliar Zn treatment using the combination of metabolome and transcriptome, and then they elucidated the molecular mechanisms underlying the foliar Zn application induced increase of 2-acetyl-1-pyrroline conferring the ‘taro-like’ aroma in pumpkin leaves. Moreover, Yue et al. used the whole-transcriptome analyses to identify key differentially expressed mRNAs, long non-coding RNAs, and microRNA associated with male sterility in watermelon. The multi-omics approach provides us a comprehensive understanding on the regulating mechanism of important traits for cucurbit crops from a systematic biology perspective.

At last, as Guest Editors, we would like to thank all the authors and co-authors for their valuable contributions to this Research Topic, all the reviewers for their important work in evaluating the submitted manuscripts. We expect this Research Topic to contribute to the advancement of genetic improvement for cucurbit crops and believe it serves as a valuable reference to our globally colleagues.

## Author contributions

QK: Writing – original draft. YZ: Writing – review & editing. JZ: Writing – review & editing. YB: Writing – review & editing.

